# Dibenzo[*a*,*e*]penta­cyclo­[12.2.1.1^6,9^.0^2,13^.0^5.10^]octa­deca-2(13),5(10)-diene

**DOI:** 10.1107/S160053681104493X

**Published:** 2011-11-05

**Authors:** Dixie Gautreaux, Tamara R. Schaller Nauman, Frank R. Fronczek, Steven F. Watkins

**Affiliations:** a Department of Chemistry, Louisiana State University, Baton Rouge, LA 70803-1804, USA

## Abstract

In the title compound, C_26_H_24_, the central cyclo­octa­tetra­ene ring has a boat conformation, and the mol­ecule is saddle shaped. The seat is defined by the mean plane of the four-atom attachment points (r.m.s. deviation = 0.014 Å) of the two bicyclo­heptane substituents. These substituents comprise the pommel and cantle, with each mean plane defined by four atoms proximate to the seat (r.m.s. deviations = 0.001 and 0.000 Å). Relative to the seat, the pommel and cantle bend up 33.36 (5) and 34.22 (4)°, while the benzo units (flaps, r.m.s. deviations = 0.008 and 0.013 Å) bend down 33.48 (4) and 36.58 (4)°.

## Related literature

For related structures, see: Cambridge Structural Database(Allen, 2002 ▶) reference codes BUPDOF and BUPDUL (Durr *et al.*, 1983 ▶) and RIBCAH (Sygula *et al.*, 2007 ▶). For the synthesis of the title compound, see: Schaller (1994 ▶).
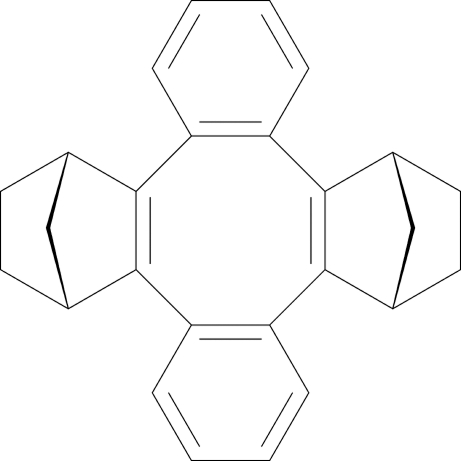


## Experimental

### 

#### Crystal data

C_26_H_24_
*M* *_r_* = 336.45
Triclinic, 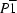

*a* = 9.6962 (4) Å
*b* = 9.7754 (4) Å
*c* = 10.3676 (4) Å
α = 77.896 (2)°
β = 63.781 (2)°
γ = 85.497 (2)°
*V* = 861.87 (6) Å^3^
*Z* = 2
Mo *K*α radiation
μ = 0.07 mm^−1^
*T* = 90 K
0.48 × 0.48 × 0.22 mm

#### Data collection

Nonius KappaCCD diffractometer
Absorption correction: multi-scan (*SCALEPACK*; Otwinowski & Minor, 1997 ▶) *T*_min_ = 0.966, *T*_max_ = 0.984
8966 measured reflections
5019 independent reflections
4347 reflections with *I* > 2σ(*I*)
*R*_int_ = 0.024

#### Refinement

*R*[*F* ^2^ > 2σ(*F* ^2^)] = 0.045
*wR*(*F* ^2^) = 0.121
*S* = 1.05
5019 reflections
236 parameters
H-atom parameters constrained
Δρ_max_ = 0.41 e Å^−3^
Δρ_min_ = −0.25 e Å^−3^

### 

Data collection: *COLLECT* (Nonius, 2000 ▶); cell refinement: *SCALEPACK* (Otwinowski & Minor, 1997 ▶); data reduction: *DENZO* (Otwinowski & Minor, 1997 ▶) and *SCALEPACK*; program(s) used to solve structure: *SHELXS86* (Sheldrick, 2008 ▶); program(s) used to refine structure: *SHELXL97* (Sheldrick, 2008 ▶); molecular graphics: *ORTEP-3 for Windows* (Farrugia, 1997 ▶); software used to prepare material for publication: *WinGX* (Farrugia, 1999 ▶).

## Supplementary Material

Crystal structure: contains datablock(s) global, I. DOI: 10.1107/S160053681104493X/mw2032sup1.cif

Structure factors: contains datablock(s) I. DOI: 10.1107/S160053681104493X/mw2032Isup2.hkl

Supplementary material file. DOI: 10.1107/S160053681104493X/mw2032Isup3.cdx

Additional supplementary materials:  crystallographic information; 3D view; checkCIF report

## Figures and Tables

**Fig. 1.**
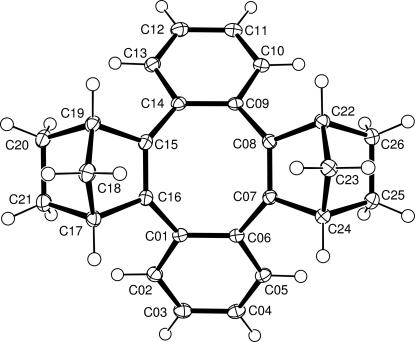
View of (I) (50% probability displacement ellipsoids)

## supplementary crystallographic information

### Comment

The central 8-ring of the title compound adopts the boat configuration, and the overall shape of the molecule is that of a saddle. Relative to the mean plane of the saddle seat (C07, C08, C15, C16, δ_r.m.s._ = 0.014 Å), the two bicycloheptane moieties (mean planes C07, C08, C22, C24, δ_r.m.s._ = 0.001 Å, and C15, C16, C17, C19, δ_r.m.s._ = 0.000 Å) bend up 33.36 (5)° and 34.22 (4)°, while the mean planes of the benzo moieties (C01 – C06, δ_r.m.s._ = 0.008. and C09 – C14, δ_r.m.s._ = 0.013 Å) bend down 33.48 (4)° and 36.58 (4)°.

### Experimental

The preparation is described by Schaller (1994). Suitable crystals were obtained by recrystallization from benzene.

### Refinement

All H atoms were placed in calculated positions, guided by difference maps, with C—H bond distances 0.95 (aromatic C) and 0.99 (alkyl C) Å, and *U*_iso_=1.2*U*_eq_, thereafter refined as riding.

### Crystal data

**Table:**

C_26_H_24_	*Z* = 2
*M**_r_* = 336.45	*F*(000) = 360
Triclinic, *P*1	*D*_x_ = 1.296 Mg m^−^^3^
Hall symbol: -P 1	Mo *K*α radiation, λ = 0.71073 Å
*a* = 9.6962 (4) Å	Cell parameters from 4596 reflections
*b* = 9.7754 (4) Å	θ = 2.6–30.0°
*c* = 10.3676 (4) Å	µ = 0.07 mm^−^^1^
α = 77.896 (2)°	*T* = 90 K
β = 63.781 (2)°	Prism, colorless
γ = 85.497 (2)°	0.48 × 0.48 × 0.22 mm
*V* = 861.87 (6) Å^3^	

### Data collection

**Table:**

Nonius KappaCCD diffractometer	5019 independent reflections
Radiation source: sealed tube	4347 reflections with *I* > 2σ(*I*)
horizonally mounted graphite crystal	*R*_int_ = 0.024
Detector resolution: 9 pixels mm^-1^	θ_max_ = 30.0°, θ_min_ = 2.8°
CCD rotation images, thick slices scans	*h* = −13→13
Absorption correction: multi-scan (*SCALEPACK*; Otwinowski & Minor, 1997)	*k* = −13→13
*T*_min_ = 0.966, *T*_max_ = 0.984	*l* = −14→14
8966 measured reflections	

### Refinement

**Table:**

Refinement on *F*^2^	Secondary atom site location: difference Fourier map
Least-squares matrix: full	Hydrogen site location: inferred from neighbouring sites
*R*[*F*^2^ > 2σ(*F*^2^)] = 0.045	H-atom parameters constrained
*wR*(*F*^2^) = 0.121	*w* = 1/[σ^2^(*F*_o_^2^) + (0.0535*P*)^2^ + 0.4358*P*] where *P* = (*F*_o_^2^ + 2*F*_c_^2^)/3
*S* = 1.05	(Δ/σ)_max_ = 0.001
5019 reflections	Δρ_max_ = 0.41 e Å^−^^3^
236 parameters	Δρ_min_ = −0.25 e Å^−^^3^
0 restraints	Extinction correction: *SHELXL97* (Sheldrick, 2008), Fc^*^=kFc[1+0.001xFc^2^λ^3^/sin(2θ)]^-1/4^
0 constraints	Extinction coefficient: 0.015 (4)
Primary atom site location: structure-invariant direct methods	

### Special details

**Table:**

Geometry. All e.s.d.'s (except the e.s.d. in the dihedral angle between two l.s. planes) are estimated using the full covariance matrix. The cell e.s.d.'s are taken into account individually in the estimation of e.s.d.'s in distances, angles and torsion angles; correlations between e.s.d.'s in cell parameters are only used when they are defined by crystal symmetry. An approximate (isotropic) treatment of cell e.s.d.'s is used for estimating e.s.d.'s involving l.s. planes.

### Fractional atomic coordinates and isotropic or equivalent isotropic displacement parameters (Å^2^)

**Table:**

	*x*	*y*	*z*	*U*_iso_*/*U*_eq_	
C01	0.28621 (12)	0.39958 (11)	−0.15111 (11)	0.0134 (2)	
C02	0.25832 (13)	0.47525 (12)	−0.26849 (12)	0.0164 (2)	
H02	0.2906	0.4377	−0.3546	0.020*	
C03	0.18494 (13)	0.60324 (12)	−0.26193 (13)	0.0182 (2)	
H03	0.1648	0.6509	−0.3415	0.022*	
C04	0.14123 (13)	0.66103 (11)	−0.13797 (13)	0.0172 (2)	
H04	0.0917	0.7489	−0.1325	0.021*	
C05	0.17053 (12)	0.58930 (11)	−0.02231 (12)	0.0153 (2)	
H05	0.1424	0.6302	0.0611	0.018*	
C06	0.24068 (12)	0.45787 (11)	−0.02517 (11)	0.01283 (19)	
C07	0.27322 (12)	0.39265 (11)	0.10023 (11)	0.01297 (19)	
C08	0.24073 (12)	0.26227 (11)	0.18361 (11)	0.01301 (19)	
C09	0.16507 (12)	0.14103 (11)	0.17577 (11)	0.0130 (2)	
C10	0.04120 (12)	0.07520 (11)	0.30452 (12)	0.0153 (2)	
H10	0.0077	0.1117	0.3914	0.018*	
C11	−0.03322 (13)	−0.04209 (12)	0.30719 (13)	0.0175 (2)	
H11	−0.1194	−0.0824	0.3940	0.021*	
C12	0.01873 (14)	−0.10005 (12)	0.18273 (13)	0.0187 (2)	
H12	−0.0311	−0.1805	0.1840	0.022*	
C13	0.14420 (13)	−0.03930 (11)	0.05642 (12)	0.0169 (2)	
H13	0.1819	−0.0814	−0.0273	0.020*	
C14	0.21718 (12)	0.08297 (11)	0.04893 (12)	0.0137 (2)	
C15	0.35046 (12)	0.14034 (11)	−0.08965 (12)	0.0137 (2)	
C16	0.37693 (12)	0.27080 (11)	−0.17235 (11)	0.0134 (2)	
C17	0.51686 (12)	0.26307 (11)	−0.31741 (12)	0.0151 (2)	
H17	0.5725	0.3538	−0.3743	0.018*	
C18	0.61003 (13)	0.15005 (12)	−0.26337 (13)	0.0171 (2)	
H18A	0.6967	0.1150	−0.3443	0.021*	
H18B	0.6465	0.1803	−0.1982	0.021*	
C19	0.47281 (12)	0.04601 (11)	−0.17980 (12)	0.0155 (2)	
H19	0.4917	−0.0432	−0.1226	0.019*	
C20	0.43547 (14)	0.03038 (12)	−0.30813 (13)	0.0179 (2)	
H20A	0.5039	−0.0380	−0.3645	0.021*	
H20B	0.3271	0.0006	−0.2711	0.021*	
C21	0.46571 (14)	0.18041 (12)	−0.40311 (12)	0.0183 (2)	
H21A	0.3713	0.2199	−0.4096	0.022*	
H21B	0.5479	0.1811	−0.5033	0.022*	
C22	0.27239 (12)	0.26323 (11)	0.31521 (12)	0.0142 (2)	
H22	0.2899	0.1695	0.3663	0.017*	
C23	0.40970 (12)	0.36642 (12)	0.24377 (12)	0.0158 (2)	
H23A	0.5024	0.3309	0.1687	0.019*	
H23B	0.4350	0.3968	0.3162	0.019*	
C24	0.32682 (12)	0.47954 (11)	0.17707 (12)	0.0146 (2)	
H24	0.3892	0.5655	0.1138	0.018*	
C25	0.18178 (13)	0.50237 (12)	0.31881 (12)	0.0170 (2)	
H25A	0.2046	0.5684	0.3676	0.020*	
H25B	0.0949	0.5382	0.2962	0.020*	
C26	0.14585 (13)	0.35287 (12)	0.41540 (12)	0.0163 (2)	
H26A	0.0416	0.3206	0.4391	0.020*	
H26B	0.1545	0.3495	0.5077	0.020*	

### Atomic displacement parameters (Å^2^)

**Table:**

	*U*^11^	*U*^22^	*U*^33^	*U*^12^	*U*^13^	*U*^23^
C01	0.0133 (4)	0.0118 (4)	0.0146 (5)	0.0001 (3)	−0.0060 (4)	−0.0017 (4)
C02	0.0187 (5)	0.0156 (5)	0.0160 (5)	0.0007 (4)	−0.0090 (4)	−0.0022 (4)
C03	0.0195 (5)	0.0166 (5)	0.0198 (5)	0.0010 (4)	−0.0116 (4)	0.0003 (4)
C04	0.0165 (5)	0.0129 (5)	0.0225 (5)	0.0023 (4)	−0.0099 (4)	−0.0017 (4)
C05	0.0150 (5)	0.0124 (5)	0.0177 (5)	0.0012 (4)	−0.0065 (4)	−0.0030 (4)
C06	0.0122 (4)	0.0114 (4)	0.0141 (4)	−0.0002 (3)	−0.0055 (4)	−0.0014 (3)
C07	0.0125 (4)	0.0125 (5)	0.0136 (4)	0.0016 (3)	−0.0053 (4)	−0.0034 (4)
C08	0.0126 (4)	0.0134 (5)	0.0129 (4)	0.0017 (3)	−0.0054 (4)	−0.0028 (4)
C09	0.0140 (4)	0.0112 (4)	0.0145 (5)	0.0016 (3)	−0.0076 (4)	−0.0009 (3)
C10	0.0165 (5)	0.0141 (5)	0.0151 (5)	0.0005 (4)	−0.0075 (4)	−0.0008 (4)
C11	0.0174 (5)	0.0149 (5)	0.0183 (5)	−0.0020 (4)	−0.0077 (4)	0.0016 (4)
C12	0.0212 (5)	0.0137 (5)	0.0222 (5)	−0.0026 (4)	−0.0111 (4)	−0.0006 (4)
C13	0.0204 (5)	0.0136 (5)	0.0182 (5)	0.0005 (4)	−0.0098 (4)	−0.0033 (4)
C14	0.0151 (4)	0.0117 (4)	0.0153 (5)	0.0017 (3)	−0.0080 (4)	−0.0019 (4)
C15	0.0145 (4)	0.0134 (5)	0.0147 (5)	0.0020 (4)	−0.0073 (4)	−0.0046 (4)
C16	0.0138 (4)	0.0142 (5)	0.0138 (4)	0.0018 (4)	−0.0068 (4)	−0.0043 (4)
C17	0.0150 (5)	0.0157 (5)	0.0141 (5)	0.0016 (4)	−0.0055 (4)	−0.0043 (4)
C18	0.0144 (5)	0.0185 (5)	0.0191 (5)	0.0034 (4)	−0.0071 (4)	−0.0063 (4)
C19	0.0169 (5)	0.0138 (5)	0.0170 (5)	0.0033 (4)	−0.0081 (4)	−0.0049 (4)
C20	0.0207 (5)	0.0164 (5)	0.0191 (5)	0.0028 (4)	−0.0096 (4)	−0.0076 (4)
C21	0.0221 (5)	0.0189 (5)	0.0157 (5)	0.0018 (4)	−0.0089 (4)	−0.0057 (4)
C22	0.0146 (4)	0.0142 (5)	0.0136 (4)	0.0006 (4)	−0.0063 (4)	−0.0024 (4)
C23	0.0149 (5)	0.0174 (5)	0.0159 (5)	−0.0007 (4)	−0.0074 (4)	−0.0027 (4)
C24	0.0156 (5)	0.0136 (5)	0.0148 (5)	−0.0002 (4)	−0.0067 (4)	−0.0029 (4)
C25	0.0191 (5)	0.0154 (5)	0.0169 (5)	0.0019 (4)	−0.0075 (4)	−0.0057 (4)
C26	0.0173 (5)	0.0169 (5)	0.0139 (5)	0.0005 (4)	−0.0058 (4)	−0.0038 (4)

### Geometric parameters (Å, °)

**Table:**

C01—C02	1.4082 (14)	C15—C19	1.5301 (15)
C01—C06	1.4099 (15)	C16—C17	1.5287 (15)
C01—C16	1.4758 (14)	C17—C18	1.5433 (15)
C02—C03	1.3891 (15)	C17—C21	1.5626 (15)
C02—H02	0.9500	C17—H17	1.0000
C03—C04	1.3914 (16)	C18—C19	1.5426 (16)
C03—H03	0.9500	C18—H18A	0.9900
C04—C05	1.3883 (15)	C18—H18B	0.9900
C04—H04	0.9500	C19—C20	1.5634 (15)
C05—C06	1.4054 (14)	C19—H19	1.0000
C05—H05	0.9500	C20—C21	1.5534 (16)
C06—C07	1.4763 (14)	C20—H20A	0.9900
C07—C08	1.3532 (14)	C20—H20B	0.9900
C07—C24	1.5314 (15)	C21—H21A	0.9900
C08—C09	1.4762 (14)	C21—H21B	0.9900
C08—C22	1.5270 (15)	C22—C23	1.5378 (15)
C09—C10	1.4082 (15)	C22—C26	1.5617 (15)
C09—C14	1.4098 (15)	C22—H22	1.0000
C10—C11	1.3910 (15)	C23—C24	1.5420 (15)
C10—H10	0.9500	C23—H23A	0.9900
C11—C12	1.3883 (17)	C23—H23B	0.9900
C11—H11	0.9500	C24—C25	1.5650 (15)
C12—C13	1.3882 (16)	C24—H24	1.0000
C12—H12	0.9500	C25—C26	1.5550 (15)
C13—C14	1.4073 (15)	C25—H25A	0.9900
C13—H13	0.9500	C25—H25B	0.9900
C14—C15	1.4779 (15)	C26—H26A	0.9900
C15—C16	1.3512 (15)	C26—H26B	0.9900
			
C02—C01—C06	118.50 (10)	C19—C18—C17	93.53 (8)
C02—C01—C16	118.22 (9)	C19—C18—H18A	113.0
C06—C01—C16	122.92 (9)	C17—C18—H18A	113.0
C03—C02—C01	121.88 (10)	C19—C18—H18B	113.0
C03—C02—H02	119.1	C17—C18—H18B	113.0
C01—C02—H02	119.1	H18A—C18—H18B	110.4
C02—C03—C04	119.48 (10)	C15—C19—C18	100.09 (8)
C02—C03—H03	120.3	C15—C19—C20	107.17 (9)
C04—C03—H03	120.3	C18—C19—C20	100.24 (9)
C05—C04—C03	119.47 (10)	C15—C19—H19	115.7
C05—C04—H04	120.3	C18—C19—H19	115.7
C03—C04—H04	120.3	C20—C19—H19	115.7
C04—C05—C06	121.88 (10)	C21—C20—C19	102.90 (9)
C04—C05—H05	119.1	C21—C20—H20A	111.2
C06—C05—H05	119.1	C19—C20—H20A	111.2
C05—C06—C01	118.76 (10)	C21—C20—H20B	111.2
C05—C06—C07	117.87 (9)	C19—C20—H20B	111.2
C01—C06—C07	123.22 (9)	H20A—C20—H20B	109.1
C08—C07—C06	130.51 (10)	C20—C21—C17	102.78 (9)
C08—C07—C24	106.88 (9)	C20—C21—H21A	111.2
C06—C07—C24	121.69 (9)	C17—C21—H21A	111.2
C07—C08—C09	131.06 (10)	C20—C21—H21B	111.2
C07—C08—C22	107.00 (9)	C17—C21—H21B	111.2
C09—C08—C22	121.23 (9)	H21A—C21—H21B	109.1
C10—C09—C14	118.74 (10)	C08—C22—C23	100.36 (8)
C10—C09—C08	117.94 (9)	C08—C22—C26	107.65 (8)
C14—C09—C08	123.18 (9)	C23—C22—C26	100.28 (9)
C11—C10—C09	121.42 (10)	C08—C22—H22	115.5
C11—C10—H10	119.3	C23—C22—H22	115.5
C09—C10—H10	119.3	C26—C22—H22	115.5
C12—C11—C10	119.87 (10)	C22—C23—C24	93.57 (8)
C12—C11—H11	120.1	C22—C23—H23A	113.0
C10—C11—H11	120.1	C24—C23—H23A	113.0
C13—C12—C11	119.37 (10)	C22—C23—H23B	113.0
C13—C12—H12	120.3	C24—C23—H23B	113.0
C11—C12—H12	120.3	H23A—C23—H23B	110.4
C12—C13—C14	121.84 (10)	C07—C24—C23	100.19 (8)
C12—C13—H13	119.1	C07—C24—C25	106.63 (8)
C14—C13—H13	119.1	C23—C24—C25	100.53 (8)
C13—C14—C09	118.66 (10)	C07—C24—H24	115.8
C13—C14—C15	118.30 (10)	C23—C24—H24	115.8
C09—C14—C15	122.96 (9)	C25—C24—H24	115.8
C16—C15—C14	129.94 (10)	C26—C25—C24	102.79 (8)
C16—C15—C19	107.15 (9)	C26—C25—H25A	111.2
C14—C15—C19	122.01 (9)	C24—C25—H25A	111.2
C15—C16—C01	131.40 (10)	C26—C25—H25B	111.2
C15—C16—C17	106.96 (9)	C24—C25—H25B	111.2
C01—C16—C17	121.07 (9)	H25A—C25—H25B	109.1
C16—C17—C18	100.22 (8)	C25—C26—C22	102.68 (8)
C16—C17—C21	107.22 (9)	C25—C26—H26A	111.2
C18—C17—C21	100.36 (9)	C22—C26—H26A	111.2
C16—C17—H17	115.6	C25—C26—H26B	111.2
C18—C17—H17	115.6	C22—C26—H26B	111.2
C21—C17—H17	115.6	H26A—C26—H26B	109.1
			
C06—C01—C02—C03	−1.34 (16)	C14—C15—C16—C17	−169.06 (10)
C16—C01—C02—C03	−174.67 (10)	C19—C15—C16—C17	0.00 (11)
C01—C02—C03—C04	1.86 (17)	C02—C01—C16—C15	−130.46 (12)
C02—C03—C04—C05	−0.52 (17)	C06—C01—C16—C15	56.53 (17)
C03—C04—C05—C06	−1.31 (16)	C02—C01—C16—C17	39.74 (14)
C04—C05—C06—C01	1.81 (16)	C06—C01—C16—C17	−133.27 (11)
C04—C05—C06—C07	177.38 (10)	C15—C16—C17—C18	−33.99 (11)
C02—C01—C06—C05	−0.48 (15)	C01—C16—C17—C18	153.68 (9)
C16—C01—C06—C05	172.52 (10)	C15—C16—C17—C21	70.33 (11)
C02—C01—C06—C07	−175.80 (10)	C01—C16—C17—C21	−102.00 (11)
C16—C01—C06—C07	−2.80 (16)	C16—C17—C18—C19	51.33 (9)
C05—C06—C07—C08	130.07 (12)	C21—C17—C18—C19	−58.47 (9)
C01—C06—C07—C08	−54.57 (16)	C16—C15—C19—C18	33.99 (11)
C05—C06—C07—C24	−37.51 (14)	C14—C15—C19—C18	−155.89 (9)
C01—C06—C07—C24	137.85 (10)	C16—C15—C19—C20	−70.13 (11)
C06—C07—C08—C09	1.38 (19)	C14—C15—C19—C20	99.99 (11)
C24—C07—C08—C09	170.36 (10)	C17—C18—C19—C15	−51.23 (9)
C06—C07—C08—C22	−168.83 (10)	C17—C18—C19—C20	58.45 (9)
C24—C07—C08—C22	0.14 (11)	C15—C19—C20—C21	67.15 (10)
C07—C08—C09—C10	−128.15 (12)	C18—C19—C20—C21	−36.87 (10)
C22—C08—C09—C10	40.89 (14)	C19—C20—C21—C17	0.10 (11)
C07—C08—C09—C14	56.15 (16)	C16—C17—C21—C20	−67.52 (10)
C22—C08—C09—C14	−134.80 (10)	C18—C17—C21—C20	36.70 (10)
C14—C09—C10—C11	−2.22 (15)	C07—C08—C22—C23	−34.13 (10)
C08—C09—C10—C11	−178.12 (10)	C09—C08—C22—C23	154.49 (9)
C09—C10—C11—C12	2.77 (17)	C07—C08—C22—C26	70.26 (10)
C10—C11—C12—C13	−0.42 (17)	C09—C08—C22—C26	−101.12 (11)
C11—C12—C13—C14	−2.45 (17)	C08—C22—C23—C24	51.37 (9)
C12—C13—C14—C09	2.95 (16)	C26—C22—C23—C24	−58.90 (9)
C12—C13—C14—C15	179.75 (10)	C08—C07—C24—C23	33.77 (10)
C10—C09—C14—C13	−0.60 (15)	C06—C07—C24—C23	−156.07 (9)
C08—C09—C14—C13	175.07 (10)	C08—C07—C24—C25	−70.56 (10)
C10—C09—C14—C15	−177.24 (10)	C06—C07—C24—C25	99.60 (11)
C08—C09—C14—C15	−1.58 (15)	C22—C23—C24—C07	−51.19 (9)
C13—C14—C15—C16	127.20 (12)	C22—C23—C24—C25	58.03 (9)
C09—C14—C15—C16	−56.14 (16)	C07—C24—C25—C26	68.46 (10)
C13—C14—C15—C19	−40.45 (14)	C23—C24—C25—C26	−35.62 (10)
C09—C14—C15—C19	136.20 (11)	C24—C25—C26—C22	−1.28 (10)
C14—C15—C16—C01	2.17 (19)	C08—C22—C26—C25	−66.56 (10)
C19—C15—C16—C01	171.23 (11)	C23—C22—C26—C25	37.88 (10)
